# The acupuncture-related therapy for post-stroke urinary incontinence: A protocol for systematic review and network meta-analysis

**DOI:** 10.1097/MD.0000000000022865

**Published:** 2020-10-30

**Authors:** Pan Cheng, Zhenhai Chi, Yuanyi Xiao, Wenping Xie, Daocheng Zhu, Ting Yu, Haiyan Li, Siyu Qin, Lin Jiao

**Affiliations:** a Jiangxi University of Traditional Chinese Medicine; b Affiliated Hospital of Jiangxi University of Traditional Chinese Medicine, Nanchang, China.

**Keywords:** acupuncture, network meta-analysis, post-stroke urinary incontinence, systematic review

## Abstract

**Background:**

With the rising incidences stroke, the Post-Stroke Urinary Incontinence (PSUI) has become one of the common clinical sequelae. PSUI not only lowers the quality of life of patients, but also impacts tremendously to mental health. As a treasure of Chinese medicine, acupuncture and its related therapies have been widely accepted in clinical treatment of PSUI. Recently, there have been many clinical studies on the treatment of PSUI with acupuncture and related therapies, but the best way to treat PSUI is controversial. Therefore, the purpose of this paper is to provide an optimal ranking regarding acupuncture and its related therapies for PSUI.

**Methods:**

The five domestic and foreign databases including PubMed, Embase, Cochrane Library, China National Knowledge Infrastructure, Wanfang Database will be systematically searched. The time range of the literature search is from the date of establishment to August 31, 2020. The main evaluation outcome was the number of patients after treatment, and the frequency of urinary incontinence. The secondary evaluation outcome was International Consultation on Incontinence Questionnaire-Short Form (ICIQ-SF), Barthel Activities of Daily Living Index (Barthel ADL Index) and the incidence rate of adverse events. The methodological quality of the article will evaluated by Cochrane Collaboration's Tool and the quality of evidence will evaluated through Grading of Recommendations Assessment, Development and Evaluation (GRADE) instrument. The Network Meta-Analysis (NMA) will be completed using Stata statistical software.

**Results:**

The final results of this study will be published in a peer-reviewed journal.

**Conclusion:**

This network meta-analysis will compare the efficacy and safety of different acupuncture therapies in the treatment of PSUI and summarize the best treatment options, which will help patients and doctors to choose effective acupuncture methods in time.

## 1 Introduction

Post-stroke urinary incontinence (PSUI) is a common disease, which is easily to be overlooked in clinical practice. The International Continence Association defines But it is agreed that urinary incontinence is one of the main sequelae after stroke urinary incontinence as urination that is not controlled by the will.^[1]^ In the past 20 years, there have been variables in research and investigation of the prevalence of PSUI in various countries, but it is agreed that urinary incontinence is one of the main sequelae after stroke. A survey based in southern China showed that the prevalence of urinary incontinence in hospitalized patients after stroke was 44.3%.^[2]^ A British study showed that 40% of 235 stroke survivors had the urinary incontinence issue within 7 to 10 days of admission.^[3]^ A longitudinal population study conducted in Australia found that 43.5% and 37.7% of stroke patients still had urinary incontinence after 3 months and 1 year respectively.^[4]^ Louis Jacob et al conducted a 10-year follow-up study on 16,181 patients who were initially diagnosed with stroke in German conventional medical institutions. Among them, the incidence of PSUI was 22% in male and 34% in female.^[5]^ PSUI has many passive effects on the patient's body and mind. Patients are prone to negative psychosocial states such as low self-esteem, conceit, embarrassment, anxiety, depression, and social isolation.^[6–8]^ It also increases the probabilities of bedsores, urinary tract infections and skin dermatitis.^[2,9]^ On the one hand, PSUI has a huge impact on patients’ quality of life.^[10–13]^ On the other hand, it brings a huge financial burden to the family. Studies have shown that the daily cost of urinary incontinence after stroke is about $185.60.^[14]^

At present, modern medicine has many treatment methods for PSUI, including pelvic floor muscle exercise (PFME), support devices, drug therapy, various electrical stimulation, surgical therapy, etc.^[15–21]^ Simple bladder training and behavior adjustment therapy are difficult to be lasting for long time, and the effectiveness is unreliable. Urinary catheter placement is prone to cause urinary tract infections, and drug treatments inevitably have side effects. Electrical stimulation and surgical treatments are invasive operations, which are expensive in medical costs and may be accompanied by complications such as pain and infection, which are poorly accepted by patients. In all, the curative effect of modern medicine in the treatment of PSUI is not ideal, so it is urgent to seek for green health, efficient and low-cost treatment methods. The acupuncture-related treatment has been commonly applied in the treatment of PSUI, and many reports have admitted the efficacy of acupuncture its related therapies in the treatment of PSUI.^[17,22–26]^ However, few studies that compare different acupuncture methods directly and it is not clear which is the best way to treat PSUI by acupuncture methods. As such, to determine the ideal acupuncture method for the treatment of PSUI is a tricky problem. This project will use NMA to evaluate and rank the integrated data, so as to provide a basis for guiding the best acupuncture treatment for PSUI.

## 2 Methods

### 2.1 Inclusion criteria for study selection

#### 2.1.1 Types of studies

All RCTs of acupuncture-related therapies for PSUI, and the language is limited to English or Chinese. Non-randomized controlled trials, clinical reviews, animal trials, individual cases, research advances, expert experience, conference articles, and duplicate articles will be excluded.

#### 2.1.2 Types of participants

Patients who have been clearly diagnosed as PSUI have no restrictions on age, gender, and race.

#### 2.1.3 Types of interventions

##### 2.1.3.1 Experimental interventions

Acupuncture will be regarded as acupoint-based therapy. (e.g., moxibustion, acupoint embedding, electroacupuncture, percutaneous electroacupuncture, auricular acupuncture, head acupuncture, acupoint injection, warm acupuncture, hand acupuncture, medium frequency electric stimulation, other comprehensive therapy, etc.), regardless of acupuncture materials, acupuncture techniques, stimulation methods.

##### 2.1.3.2 Control interventions

The control group will take rehabilitative treatment. Research on different types of acupuncture methods will be included.

#### 2.1.4 Types of outcome measurements

##### 2.1.4.1 Primary outcomes

(1) Number of patients after treatment.
(2) Frequency of urinary incontinence (including total and average episodes).

##### 2.1.4.2 Additional outcomes

(1) International Consultation on Incontinence Questionnaire-Short Form (ICIQ-SF).
(2) Barthel Activities of Daily Living Index (Barthel ADL Index).
(3) The incidence rate of adverse events.

### 2.2 Literature search

The data involved in this research are all searched by computers in PubMed, Embase, Cochrane Library, China National Knowledge Infrastructure, Wanfang Database. The search strategy adopts subject terms and The method of combining free words, adjust the search terms according to the search results, and the search time is from the establishment of the database to August 31, 2020. The retrieval strategy of PubMed is shown in Table 1.

**Table 1:**
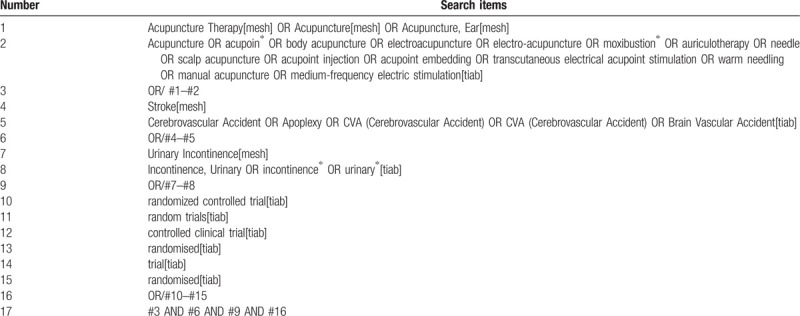
Search strategy used in PubMed database.

### 2.3 Data collection and analysis

#### 2.3.1 Selection of studies

**Figure 1:**
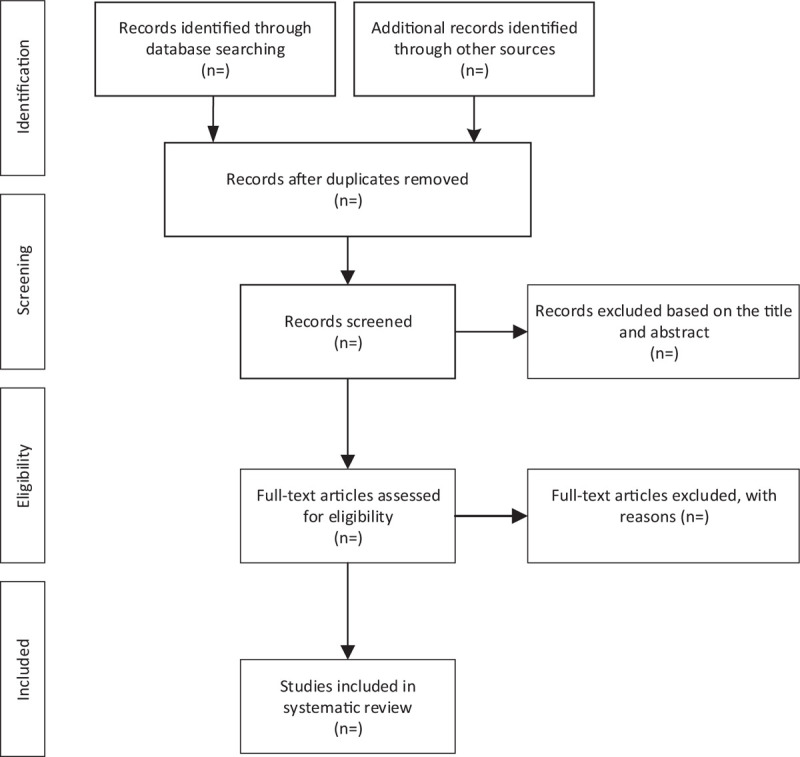
Flow diagram of study selection process.

We will use the EndNoteX7 tool to import the literature retrieved from the five databases. First, we used software to delete duplicate articles, then 2 reviewers independently browsed the title and abstract, and deleted the articles that did not meet the requirements. If they cannot judge whether it is a qualified study, they need to read the full text to decide. After that, the two reviewers will cross-check whether the final selected research is consistent, and if there is a difference, it will be decided through a group discussion. The research selection process is shown in Figure 1.

#### 2.3.2 Data extraction and management

We will use Microsoft Excel 2018 to build an information data extraction table and perform pre-extraction to determine the feasibility of the table. The extracted data will mainly include the following information: basic information (title, first author, country, publication year), patient characteristics (gender, age, number of persons, country, diagnostic criteria), methodological information (grouping method, allocation concealment, blind method, result bias), intervention measures (treatment measures, treatment time, frequency), results (data of primary and secondary results). The data extraction was completed independently by two researchers, and then the results were cross-checked. If there were inconsistent results, the final results were determined through group discussion.

#### 2.3.3 Assessment of risk of bias in included studies

The two researchers strictly followed the Cochrane Manual to independently evaluate the article methods of the selected studies, and then to ranked the included literatures from the following aspects: the random sequence generation, blind (or mask), result evaluation, allocation concealment, and incomplete data evaluation, selective reports and other sources of bias. Any disagreement from the ranking, would be resolved by the third reviewer.^[27]^

### 2.4 Data synthesis

#### 2.4.1 Management of lost data

If there is insufficient data from the selected study, we will send an email to try to contact the author and obtain the complete data. If the baseline outcome data or other data were included, we can use Cochrane to manually calculate the mean and standard deviation of the changes.

#### 2.4.2 Data synthesis and statistical methods

Before data synthesized, a heterogeneity test was performed on the included studies to check whether the included articles could be merged.^[28]^ If I^2^ ≤ 50%, a fixed-effect model will used for analysis. Instead, the data will be processed using a random effects model. Effects of continuous variable data were expressed by standardized mean difference (SMD) and associated 95% confidence intervals (CI). Hazard ratio and associated 95% CI to express the effects of categorical variable data. For direct comparisons, traditional two-by-two meta-analyses were used, while indirect comparisons used network meta-analyses. For data analysis, we will use R software 3.6.1 and the related “NetMeta” software package to complete. The evaluation of the inconsistency between the direct and indirect comparison results will use the Z-test, and the results will be represented by a network graph. If there was high intertrial heterogeneity, the subgroup analysis is also required. In addition, The funnel plots and Egger regression tests will also be used to detect publication bias.^[29]^

#### 2.4.3 Grading the quality of evidence

According to the criteria in the GRADE system, the quality of the study was evaluated by two authors and was classified into four grades: “high”, “medium”, “low” and “very low”, and the results were then exchanged. If there was any disagreement, the final proposal would be selected through group discussion.^[30]^

## 3 Discussion

Clinically, acupuncture-related therapy has been widely used in the treatment of PSUI, but it is short of direct comparison among clinical curative effects, as such, the best method of acupuncture-related therapy to treat PSUI is hard to be chosen for doctors or patients. This network meta-analysis is to evaluate the curative effect and safety by different acupuncture-related therapies for PSUI, it will have direct and indirect evidences via network meta-analysis, the purpose is to provide a ranking for the acupuncture-related therapy of PSUI. However, there were potential limitations to this study. For one thing, only Chinese and English literatures were included in this study, so incomplete included data may cause deviation to the research results. For the other thing, the quality of the original trial will affect the reliability of the pooling effect, so we will strictly control the quality of the included studies.

## Author contributions

**Data curation:** Pan Cheng, Yuanyi Xiao

**Investigation:** Zhenhai Chi, Siyu Qin

**Methodology:** Daocheng Zhu, Ting Yu

**Resources:** Lin Jiao

**Software:** Haiyan Li, Wenping Xie

**Writing – original draft:** Pan Cheng, Yuanyi Xiao

**Writing – review & editing:** Pan Cheng, Lin Jiao
